# Parental presence during invasive pediatric procedures: what does it depend on?^1^

**DOI:** 10.1590/1518-8345.6101.3828

**Published:** 2023-03-06

**Authors:** Laura Palomares González, Iván Hernández Caravaca, Carmen Isabel Gómez García, Manuel Sánchez-Solís de Querol

**Affiliations:** 1 Virgen de la Arrixaca University Children’s Hospital, Department of Neonatal ICU, El Palmar, Murcia, Spain.; 2 University of Alicante, Department of Community Nursing, Preventive Medicine and Public Health and History of Science, Alicante, Comunidad Valenciana, Spain.; 3 University of Murcia, Faculty of Nursing, Murcia, Murcia, Spain.; 4 Virgen de la Arrixaca University Children’s Hospital, Department of Pediatrics, El Palmar, Murcia, Spain.

**Keywords:** Child Care, Parenting, Attitude of Health Personnel, Pain, Procedural, Surveys and Questionnaires, Word Processing, Cuidado da Criança, Poder Familiar, Atitudes do Pessoal de Saúde, Dor Processual, Inquéritos e Questionários, Processamento de Texto, Cuidado del Niño, Responsabilidad Parental, Actitud del Personal de Salud, Dolor Asociado a Procedimientos Médicos, Encuestas y Cuestionarios, Procesamiento de Texto

## Abstract

**Objective:**

family-centered care during invasive procedures has been endorsed by many professional health care organizations. The aim of this study was to evaluate the health professionals’ attitudes towards parental presence during their child’s invasive procedure.

**Method:**

pediatric healthcare providers (divided in professional categories and range of ages) from one of the Spain’s largest hospitals were asked to complete a questionnaire and write free-text comments.

**Results:**

227 responded the survey. Most (72%) participants, in their answers, reported that parents are sometimes present during interventions, although there were differences between professional categories in this respect. The procedures in which the parents were present were those considered “less invasive” (96% of cases), while only 4% were present in those considered “more invasive”. The older the professional, the less necessary parental presence was considered.

**Conclusion:**

the attitudes towards parental presence during pediatric invasive procedure are influenced by the professional category, the age of the healthcare provider and the invasiveness of the procedure.

## Resumo

**Objetivo:**

o atendimento centrado na família durante procedimentos invasivos tem sido endossado por muitas organizações profissionais de saúde. O objetivo deste estudo foi avaliar as atitudes dos profissionais de saúde em relação à presença dos pais durante o procedimento invasivo realizado em seus filhos.

**Método:**

os prestadores de serviços de saúde pediátricos (divididos em categorias profissionais e faixa etária) de um dos maiores hospitais da Espanha foram solicitados a preencher um questionário e escrever comentários de texto livre.

**Resultados:**

a pesquisa foi respondida por 227 pessoas. A maioria (72%) dos participantes, em suas respostas, relatou que os pais algumas vezes estão presentes durante as intervenções, embora houvesse diferenças entre as categorias profissionais a esse respeito. Os procedimentos em que os pais estavam presentes eram aqueles considerados “menos invasivos” (96% dos casos), enquanto apenas 4% estavam presentes naqueles considerados “mais invasivos”. Quanto mais velho o profissional, a presença dos pais foi considerada menos necessária.

**Conclusão:**

as atitudes em relação à presença dos pais durante o procedimento pediátrico invasivo são influenciadas pela categoria profissional, a idade do prestador de serviço de saúde e a invasividade do procedimento.

## Resumen

**Objetivo:**

la atención centrada en la familia durante procedimientos invasivos ha sido respaldada por muchas organizaciones profesionales dedicadas al cuidado de la salud. El objetivo de este estudio fue evaluar las actitudes de los profesionales de la salud con respecto a la presencia de los padres durante los procedimientos invasivos realizados en niños.

**Método:**

a los prestadores de atención médica en Pediatría (divididos en categorías profesionales y rangos de edad) de uno de los hospitales más importantes de España se les solicitó que respondieran un cuestionario y redactaran comentarios de texto libre.

**Resultados:**

un total de 227 profesionales respondieron la encuesta. En sus respuestas, la mayoría (72%) de los participantes informó que, en ocasiones, los padres están presentes durante las intervenciones, aunque se registraron diferencias entre las distintas categorías profesionales al respecto. Los procedimientos en los que los padres estuvieron presentes se consideraron como “menos invasivos” (96% de los casos), mientras que solamente el 4% estuvo presente en los considerados “más invasivos”. A mayor edad de los profesionales, menos necesaria se consideró la presencia de los padres.

**Conclusión:**

las actitudes con respecto a la presencia de los padres durante procedimientos pediátricos invasivos se vieron influenciadas por la categoría profesional, la edad del prestador de salud y la invasividad de los procedimientos.

**Highlights**

(1) Parental presence during pediatric invasive procedures is still a controversial topic.

(2) The age of the professional influences the attitudes towards parental presence.

(3) Procedure invasiveness modify professionals’ attitudes towards parental presence.

(4) Innovative text analysis was applied to analyze health professionals’ comments.

(5) Protocols and specific training are widely demand to include parent in child’s care.

## Introduction

Until the first half of the 20^th^ century, children were systematically separated from their parents during hospitalization. The psychoanalysts René Spitz and John Bowlby strongly contributed to our understanding and knowledge of the psycho-affective consequences of this parental deprivation for young infants, defining it as “hospitalism” and proposing the “attachment theory”, respectively^1^ ^-^ ^4^. These authors initiated the trend from the traditional paternalistic model of medicine towards family-centered care^5^, which involves a greater degree of partnership between the family and health care providers^1^ ^,^ ^6^ ^-^ ^8^.

Invasive pediatric procedures can be painful and frightening experiences for children and their parents. In the European Charter for hospitalized children (1986), it is established that it is a child’s right to be accompanied by their parents throughout the hospitalization process, with parents becoming an active part of hospital life^9^. In addition, parents’ interest in being present when procedures are performed has increased over the years^10^. Many publications demonstrate the desire of parents to accompany their children during the performance of painful procedures such as venipunctures, placement of venous accesses, urethral catheterization or performing a lumbar puncture and it presence is beneficial for both family members and health care staff^11^. There is significant evidence that the parental presence during invasive procedures can reduce a child’s anxiety and pain, while accelerating the recovery process in children and reducing the anxiety of family members^12^ ^-^ ^14^. Moreover, different medical and nursing associations have all put forward recommendations or resolutions concerning the presence of family members during invasive procedures and, if necessary during resuscitation in the hospital^15^ ^-^ ^17^.

However, some of the healthcare professionals are less willing to allow such parental presence despite it being the child’s right. The two main reasons expressed by the care providers in this respect include parental anxiety and the greater nervousness on the part of children. Other reasons are related with the time required to explain the procedure to parents and the healthcare providers’ anxiety for the parental presence during the intervention^18^. Some authors suggest undeniable evolution in the professionals’ opinions concerning parental presence^19^. In most cases, the attitudes of health professionals are evaluated through questionnaires because of the difficulty involved in analyzing free texts.

The aim of this study was to evaluate the health professionals’ attitudes towards parental presence during their child’s invasive procedure.

## Method

### Type of study and localization

This is a cross-sectional study, guided by the STROBE (Strengthening the Reporting of Observational Studies in Epidemiology) guideline, used to report observational studies^20^. The study was performed by means of an anonymous self-administered questionnaire in a large University hospital in Murcia, Mur, Spain.

### Period

The data collection period was between October 3^rd^, 2019 and June 30^th^, 2020.

### Population and selection criteria

The hospital employs 4000 health professionals^21^, who are responsible for providing hospital care for 250,000 people, 133,000 of them are pediatric population (from 0 to 14 years old) according to the Spanish National Statistical Institute**.** Pediatric population are defined in Spain as children from 0 to 14 years old although this range vary between countries^22^.

Workers from the pediatricians, nursing and nursing assistant teams (pediatrician, pediatrician resident, pediatric nurse, pediatric nurse resident and nursing assistant) belonging to eleven different pediatric units (Emergency, Intensive Neonatal Care, Intensive Pediatric Care, Oncology, Neonatology, Infant hospitalization, Pre-school hospitalization, School-age hospitalization, Surgery, Reanimation and Ambulatory pediatrics) in the hospital were invited to the study. 

### Sample definition

All the workers of pediatric units were invited to the study. In order to avoid bias a minimum sample size base on the total eligible population (n=444) was calculated using the statistical software SAS University Edition. To determine the minimum sample size of responders with statistical relevance, a 75% rate of parental presence with a precision of 5% and a confidence level of 95% was estimated based on previous studies in Spain^23^. The resulting minimum sample size was 175. The final responders obtained in our study were 227, countersigning the robustness of our results. This sample size was selected after dividing into professional categories and according to pediatric units.

### Instruments used to collect the information and study variables

An anonymous self-administered questionnaire, which consisted of 14 multiple response questions were used. The questionnaire was based on a published questionnaire^18^, originally developed in Spanish to access the parental presence during invasive procedures in the perspective of those responsible for Pediatric Emergency Services and active members of Spanish Society of Pediatric Emergencies.

The questionnaire contained sociodemographic information (gender, age), professional category (pediatrician, pediatrician resident, pediatric nurse, pediatric nurse resident and nursing assistant) and pediatric units. 

The survey covered variables such as parental presence during invasive pediatric procedures (yes, yes sometimes, no), the type of procedures performed which parents were allowed to be present (eleven invasive procedures could be selected), the reasons for any reluctance to allow parental presence (related to parents, to the child, interruption of the procedure or aggression and claims) and an evaluation of the existence for protocols and specific training regarding parental presence (yes, no). Moreover, a Likert scale were used to determine the frequency of the problems derived from the presence of the parents (never, occasionally, frequently, always). After completing the questionnaire, participants were invited to write free-text comments, motivated by the sentence: “We would appreciate any comment on the subject”. Eleven invasive procedures were included in the study and divided according to the degree of invasiveness, into less and more invasive (Figure 1).

**Figure 1:** Procedures included in the questionnaire by invasiveness. Murcia, Mur, Spain, 2020

Invasiveness	Procedure
Less invasive	Nasal wash
	Venipuncture
	Peripheral venous canalization
	Bladder catheterization
	Oro-nasogastric catheterization
	Wound healing or suturing
More invasive	Lumbar puncture
	Central or arterial canalization
	Skeletal traction
	Endotracheal intubation
	CPR maneuvers

### Data collection

The workers were invited through the institutional email service. After that, printed questionnaires and informed consent form were sent to each pediatric unit. As a strategy during data collection, visits were made to all of the units to invite and instruct the professionals to participate in the research.

### Data treatment and analysis

The questionnaires received were processed using Microsoft Excel, and statistical analysis was performed using the statistical software SAS University Edition. The presence/absence of parents during invasive procedures with their children, the age ranges of healthcare professionals (<40, 40-50, >50 years), and the proportion of parents present in “more” or “less” invasive procedures were analyzed using Pearson’s χ^2^ analysis. A multivariate analysis (Fisher logistic regression) was explored considering presence/absence of parents as dependent variable and professional category and age as independent variables. 

The free-text comments in the questionnaires were evaluated by a text analyst program KH coder^24^, which produces a list of words ordered according to their frequencies and interrelationships to analyze and visualize the text content as a co-occurrence network. Frequently co-occurring terms in the visualization are connected by lines/edges: the relative frequency of terms is indicated by the relative size of their node, and the relative frequency of co-occurrence of terms is indicated by the relative thickness of the edge connecting their nodes.

### Ethical aspects 

This study was carried out in accordance with the ethical standards established by the Helsinki Declaration (2000) and Istanbul Declaration (2008).

Health care professionals were invited to answer the questionnaire after being informed of the confidentiality of the survey according to the European General Data Protection Regulation 2016/679 and allowed sufficient time to read it.

## Results

### Analysis of questionnaires

Two hundred and twenty-seven out of 444 questionnaires were received (51.1%) completed by 196 female (86.34%) and 31 male (13.65%) participants from 11 different pediatric services. 

The respondents’ ages were aggregated and balanced into three groups <40 years old, from 40 to 50 and >50 years (40%, 26% and 34% respectively). The respondents were also placed into 3 professional categories: 1. Pediatrician and pediatric residents (n=50); 2. Pediatric nurses and pediatric nurse residents (n=109) and 3. Nursing assistants (n=68).

**Figure 2:**
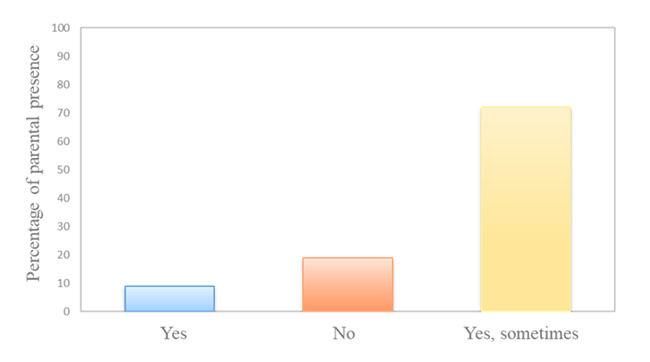
Percentage of respondents answering Yes (blue bar), No (red bar) and Sometimes (yellow bar) concerning parental presence during invasive pediatric procedures (p<0.0001). Murcia, Mur, Spain, 2020

In the first part of the questionnaire, the presence of parents in the different units was investigated for the 227 respondents. Most (72%) reported that parents were sometimes present during interventions, 19% never performed procedures in the presence of parents and 9% reported that parents were always present during procedures (p<0.0001) (Figure 2).

**Figure 3:**
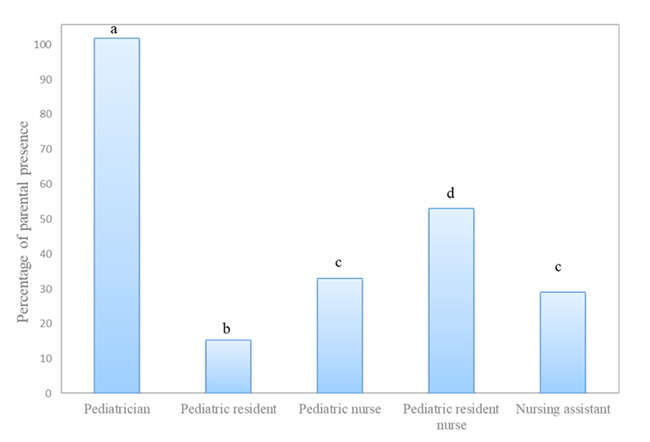
Percentages of respondents in favor of the presence of parents in the different health professional category. Different letters a, b, c and d indicate significant differences in the affirmative responses between the different professional categories (p <0.05). Murcia, Mur, Spain, 2020

No differences were found when these results were analyzed taking into account the professional category of the respondents. However, there were differences according to professional category when only the professionals that always or never performed procedures in the presence of parents were analyzed. The percentages in this respect were that pediatricians reached 100% of presence, followed by pediatric nurse residents (50%), pediatric nurses (31%), nursing assistants (27%) and pediatric residents (14%) (p<0.05) (Figure 3).

In terms of the type of procedures where parents were allowed to be present, the percentages were 96% in the case of “less invasive” and 4% in the case of “more invasive” (p<0.0001). 

In regard to any problems derived from parental presence, 61% of the professionals reported occasional problems (n=122), 29% had never had problems (n=58) and 10% said they frequently had problems (n=18). The reported problems related to such presence were: greater nervousness on the part of child (37%, n=83), parent’s indisposition (example: dizziness) (35%, n=80), interrupting the procedure (22%, n=49) and others such as aggression or complaints (7%, n=17).

Most professionals thought that parental presence was unnecessary and preferred to perform procedures without them (72% n=164), while 28% (n=53) thought such presence was necessary. The main reasons stated for the reluctance to allow parents to be present were parental anxiety (65%), the belief that the parents are not prepared to stay (41%), the invasiveness of the procedures (39%), reduced physical space (23%), the idea that they would perform less well (20%) and greater nervousness on the part of child (18%).

**Figure 4:**
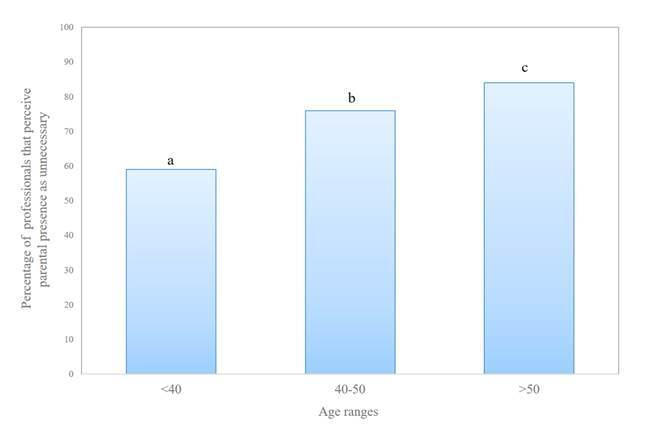
Percentage of professionals that perceive parental presence as unnecessary according to age ranges. Different letters a, b and c indicate significant differences between the different range ages, (p = 0.004). Murcia, Mur, Spain, 2020

Most of the professionals in group aged >50 perceived as unnecessary the present of the parents showing the highest percentage of negative answers (84%). Although there were differences between the three subgroups (76% in the group aged 40 to 50 years, and 59% of negative answers for those aged <40 years) (p=0.004) (Figure 4).

When the “Yes” or “No” answers concerning the need for parental presence were compared within the same age range, statistically significant differences were found within the 40 to 50 group and >50 group (p = 0.0004 and p <0.0001, respectively).

Furthermore, 82% of the professionals indicated that there were no written protocols related to parental presence in the different pediatric units, while the need to create them and to receive relevant training was supported by 75% of those interviewed. Statistical differences in terms of the need of training were found between group <40 (83%) and the groups 40 to 50 (66%) and >50 group (66%) p<0.005. 

### Quantitative free-text analysis

Fifty-nine respondents wrote comments in the free-text part of the questionnaire. Analysis of their comments showed that 1435 words were used in a total of 75 sentences. The most frequent words were the verb “be” (101 times), followed by “parent” and “child” (74 and 35 times, respectively), the adverb “not” (35 times), “procedure” (26 times), and the noun “presence” (24 times). 

**Figure 5:**
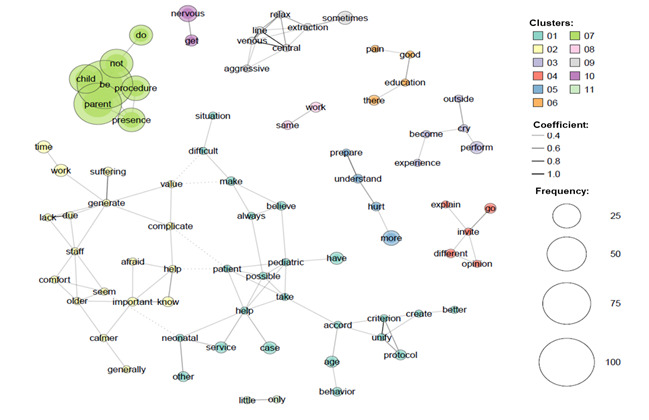
The co-occurrence network of words identified 11 clusters. Frequently co-occurring terms in the visualization are connected by lines/edges, the relative frequency of terms is indicated by the relative size of their node, and the relative frequency of co-occurrence of terms is indicated by the relative thickness of the edge connecting their nodes. Murcia, Mur, Spain, 2020

The results of the co-occurrence network of words analysis revealed 11 clusters regarding how the professionals evaluated parental presence during invasive pediatric procedures (Figure 5). 

The most important cluster in terms of frequency of the words used and the thickness of it edges was cluster 7 represented in green in Figure 5. This cluster contained the words “be, parent, do, not, procedure, child, presence” and some examples of sentences using these words are: “Parents should not be present during invasive procedures”, “I think it depends a lot on the type of parents and the child, it cannot be generalized” or “I don’t think it’s good for the child to associate parents with pain in a procedure”.

Another important cluster is number 10 (in purple), which matched the words “get” and “nervous” as exemplified by the sentences: “I think that parents should not be present, they get very nervous” or “I do not agree that the parents be present during procedures, they get very nervous”.

Cluster 5 (in blue) brought together “prepare”, “hurt”, “understand” and “more”, as in the comments: “Children are more restless (they do not understand that they are hurting them and their parents do not defend them), professionals feel supervised and most families are not prepared”. “I think that in some techniques some parents are not prepared to understand what we do”.

Another cluster relates to the performance of professionals (number 3, colored light purple in Figure 5) and include words like: “perform”, “experience”, “become”, “cry” and “outside”. One example of a sentence included in this cluster is “When a procedure is performed on the child, the parents should not be there because if the child cries, the parents become nervous, it is better to do it without them”.

## Discussion

In our study, the vast majority of the pediatricians, nurses, and nursing assistants (72%) said that they sometimes perform invasive procedures in children with parental presence. Nineteen percent of them claimed that they never perform invasive procedures with parental presence and 9% affirmed that they always performed procedures in the presence of the parents.

There were differences between the different professional categories concerning the answers “never” and “always”. Pediatricians performed more procedures with parental presence than pediatric residents (100% and 14%, respectively). Furthermore, previous studies in Europe showed that residents from various specialties more often prefer paternalistic decision making than their supervisors. They suggest that this might be attributed to differences in the degree of professional confidence and their lack of knowledge about shared decision making^25^. Pediatric residents could show lack of confidence compared with the skills of more experienced pediatricians. However, a study found little difference between the satisfaction of both groups concerning the presence of parents during an intervention^23^. In our study, we also found differences between the attitudes of different nursing groups. Contrary to the findings for pediatricians, in the nursing category the group that performed more procedures with parental presence were the pediatric nurse residents as opposed to pediatric nurses (50% *vs.* 31%). Our results agree with those described in other Spanish studies, which showing pediatricians to be more in favor to parental presence than nurses, with those that disagree most being pediatric residents^26^ ^-^ ^27^. The lower values found for the pediatric residents compared to pediatric nurse residents might be attributed to the difference in the length of the practicums during their respective degree courses, and to the fact that nurses are focus on the procedures during their practicums. In contrast to our study, others European and American studies found that nursing professionals are most in favor of family presence during the performance of invasive procedures. On the other hand, no differences were found between nurses and pediatricians in some countries of Asia^28^ ^-^ ^31^. These results could also be explained in our context because, most of the pediatric nurses were unable to get their specialty in pediatric care because the pediatric specialty is relatively young in Spain. However, all other professional categories were able to get it or were undergoing pediatric training. 

In regard to the wishes of parents to be present during invasive procedures even in worse-case scenarios such as critically ill children or resuscitation, they want to be there^32^ ^-^ ^35^. It has been also reported that families perceive that their presence is helpful to the patient and essential for medical care^36^. They need to feel part of, and be involved in, their child’s care and their presence increases their sense of control, helps them to cope with the stressful situation and the satisfaction and relationship with health-care providers^6^ ^,^ ^37^ ^-^ ^39^. Although evidence for the use physical (*e.g.,* breastfeeding, kangaroo/maternal holding, nonnutritive sucking, white noise in new-born^38^ ^-^ ^40^), and psychological (*e.g.*, clown therapy, distraction^41^ ^-^ ^45^) interventions to manage procedural pain has been widely advocated in the published literature, and is the family the driver of many of them, the implementation in practice sometimes is lacking^46^ ^-^ ^47^. In our study, parents were allowed to be present in 96% of “less invasive” interventions, and only in 4% in the case of “more invasive” procedures. Also, other authors have demonstrated that the more invasive the procedures are, the less professionals want to have parents present^48^. 

Few problems regarding parental presence during invasive procedures were reported by the respondents. Most of the providers (90%) mentioned occasional (61%) or no problems (29%) had arisen during a given procedure, coinciding with other study, which found a high level of satisfaction (57%) of the professionals when an invasive procedure is performed in the presence of parents. Furthermore, in the same study, 90% of family members and 76% of professionals believed that it had been beneficial for the child^23^ and considered the family as a resource in nursing care^31^. 

The main problems mentioned by professionals in our study concerning parental presence were, greater nervousness on the part of child (37%), parent´s indisposition (35%), procedure interruption (22%) and aggression or complaints (7%). These problems have been reported in another study, in which the most frequently mentioned problems were dizziness (78.6%), greater nervousness on the part of child (63.3%), interruption of the procedure due to the parents (anxiety, interference in the procedure) (46.4%) and finally complaints or reclamations (8%)^18^. Some of these problems reported can be manage by a correct family-centered care approach. That means not just allow parental presence but make recommendations regarding current assessment tools, parenting behavior interventions for reducing pediatric procedural distress^49^ and even the follow-up of the treatment recommendations after discharge^50^ ^-^ ^51^. An active role can be achieved by parents with timely, relevant information and could also help them feel in control of the situation, and relieves their own stress^52^ ^-^ ^53^. Both parents and healthcare providers have a role in reducing the distress and pain that children experience during necessary medical procedures and none of them have to be eliminated in this paramount purpose. 

In our study, 65.4% of the 227 professionals did not consider the presence of the parents to be necessary. Regarding that, an innovative free-text analysis was used for the first time to evaluate the comments on parental presence wrote by healthcare providers, although KH Coder have been used in studies of public health^54^ ^-^ ^55^. This analysis demonstrated the increased understanding of people’s attitude to a given topic^54^ ^,^ ^56^ and revealed that the most important cluster (number 7) used words such as “not”, “parent”, “presence” and “procedure”. This analysis is in accordance with the result of the questionnaire in which more than a half of the responders are contrary to the need of parental presence. Similar figures to ours were obtained in an study carried out in the emergency service of a Spanish hospital, in which only 60% of the providers approved of the presence of the parent during less invasive procedures, and only 10.8% would encourages the family’s presence during resuscitation^57^. The views of pediatric nurses concerning parental presence during painful procedures, evaluated in a Turkish study, stated that 56.3% of the nurses thought that parents should not be present during invasive procedures^29^. However, higher figures of parental presence during anesthesia induction (86.9%) were obtained when nurse anesthetist were asked in a nationwide survey in Sweden^31^.

The present study notes that the reasons given for any reluctance to allow parental presence are mainly parent-related, including parental anxiety (65%), belief that parents are not prepared to stay (41%), the invasiveness itself of the procedure (39%), reduced physical space (23%), poorer care performance (20%) and greater nervousness on the part of child (18%). In the case of parental anxiety, these results were reinforced in the free text analysis, where cluster number 10 associated words like “get” and “nervous”. The belief that parents are not prepared was represented in the free text comments by cluster number 5 with words like: “prepare” and “understand”. Somehow the clusters 10 and 5 can be associated with the theme “Not prepared for the experience” that was reported by some healthcare providers in face-to-face, in-depth, semistructured individual interviews in a qualitative study in South Africa^58^. Otherwise, the invasiveness of the procedure and poorer provider performance can be seen in cluster number 3 that matched words like “perform”, “experience”, “become”, cry” and “outside” and could fits with the theme “A traumatic experience” in the same study”^58^.

Parental anxiety was also given for reluctance to allow parental presence in another study carried out in an emergency pediatric service (86% of the respondents), being similar to our results. By contrast, other explanations were more frequent: fear of poorer staff performance (77%), belief that parents are not prepared to stay (76%), the invasiveness of the procedure (70%), and increase child anxiety (39%)^18^. Maintaining self-control during their child’s procedure was important for parents and represented a significant source of stress. For some parents, emotions (*e.g.*, a fear of needles) interfered with their ability to be present during a procedure, making them feel guilty or that they were not fulfilling their parental role. In some cases could be the “important others” (families of parents or friends) the ones who help and support the pediatric invasive procedure^59^. Thus, parental preference varies, that´s why an open line of communication with healthcare providers and acceptance of parents’ views can support participation and improve child safety^60^.

There is a vast quantity of scientific information concerning the benefits of parental presence during invasive pediatric procedures^12^ ^-^ ^14^, based on which findings some hospital have proposed the figure of family presence facilitator. This person would be charged with giving proper explanations of the procedure to parents, which would be easier and faster manner of involving them in their child’s care^61^. The family presence facilitator would work with both parents and health professionals. We are of the opinion that such a post would be worth creating in our and other hospitals to reduce any reluctance to accepting the presence of a parent. 

In reference to the ages of professionals and their views concerning necessity of parent presence, our study found that the older the care provider the less parental presence was considered beneficial (59% of negative answers in the <40 years old group, 76% in 40 to 50 years group and 84% in the >50 years old group, P=0004). A possible explanation in our context would be the rare negative experiences the professionals had had during their careers and the lack of formal training in pediatrics in the case of nursing assistants and pediatric nurses. Similar results are described in studies from Brazil and South Africa, in which parental presence was more widely accepted by professionals with less experience^62^ ^-^ ^63^. However, such differences were not found in a large Asian study concerning professional experience or age and the preference for parental presence. In the study^48^ more than 90% of the pediatricians and nurses preferred to perform procedures without the parent’s presence.

Parents want to be present during invasive procedures and they recommend being present to support increased coping for their children^37^. However, parents may suffer high anxiety levels in the face of invasive procedures, which could perhaps be reduced if suitable information about what is going happen in a particular procedure. Without such information a parent´s anxiety could have a negative effect on their children^64^. Despite that, the fact that 65% of the healthcare providers in our study did not consider the presence of the parents to be necessary, we found a high percentage of demand for written protocols to be adhered to during professional service (82% of professionals) and trainings (75%) on parental presence showing that they need professional tools and scientific support to fully implement family centered care within their services. Without a clear understanding of family care management, the concept is at risk of being relegated to a vague colloquial expression^65^. Moreover it has been seen that permitting a parent’s presence and changes in traditional views might occur if the professionals had protocols and were properly trained^36^.

The findings of our study could also help to determine which groups need to receive such training in the first place. Defining roles within work teams could speed up not only parental presence during invasive procedures but also decrease children’s pain, stress and negative behavior in general during invasive procedures^14^. This would also improve the professional comfort during procedures and well-being of the entire family^61^ ^,^ ^66^. To successfully implement family-centered care, a shift in healthcare provider caregiving practices is necessary to promote mutual respect, collaboration, and support for parents. To create such protocols, a multidisciplinary team is required to identify, coordinate and address shared goals that meet the needs of the child. The figure of a family presence facilitator could be included in such protocols, as their role would promote the implementation of family-centered care. Such a person would reinforce explanations of the procedure and coordinate with health professionals, encouraging them to include parents in the task of child care during the invasive procedure. 

The present study brings us the opportunity to know what is behind parental presence or not within one of the largest hospitals in the south-east of Spain, but this could also be a limitation of our study, since it really reflects the opinion of the professionals of this hospital. Another limitation could be the lack of a pure qualitative study. Future research with individual interviews or even through focal groups may be a future path for a better understanding of the object and scenario studied. 

The contribution of this study allows us to know the perspectives of healthcare professionals about a very important subject. In most of the countries, where family-centered care in more implemented, exist internal policies to be follow by the professionals that allow parents to be present in all procedures. Thus, in these cases the attitude of practitioner is less important. However, in our context, parental presence is determined by the professional´s attitude. According to our study findings, there is a demand for written protocols or guidelines and specific trainings. This could speed up the acceptance of parental presence during invasive procedures. Moreover, the main reason stated for the reluctance to allow parents to be present were parental anxiety. This can be management with timely and relevant information to make parental interventions an important tool for professionals^67^ and not a problem to increase child´s well-being and safety during invasive procedures.

## Conclusion

The results showed that the attitudes towards parental presence during invasive pediatric procedures are influenced by the professional category, age and the degree of invasiveness of the procedure. Moreover, more than half of the respondents did not perceive the presence of a parent as being necessary when invasive procedures are being performed. The rare problems reported when parents were present during invasive procedures were mainly related with greater nervousness on the part child, the parent’s indisposition, and interruption of the procedure. The findings show that there is a widely felt demand for written protocols and specific training in the different medical units to clarify how best to include parents in child´s care during invasive procedures.
